# Help and support for gambling harm among United Kingdom Armed Forces personnel: A mixed-methods study

**DOI:** 10.1177/13558196251409041

**Published:** 2025-12-20

**Authors:** Blair Biggar, Hannah Champion, Matthew Jones, Glen Dighton, Justyn Larcombe, Matt Fossey, Simon Dymond

**Affiliations:** 1 School of Psychology, 7759 Swansea University, Swansea, UK; 2 School of Social and Political Studies, 3526 University of Glasgow, Glasgow, UK; 3 Centre for Military Gambling Research, 7759 Swansea University, Swansea, UK; 4 The Recovery Course, Tonbridge Baptist Church, Tonbridge, UK; 5 Veterans’ and Families Institute, 2369 Anglia Ruskin University, Chelmsford, UK; 6 Department of Psychology, Reykjavík University, Reykjavík, Iceland

**Keywords:** gambling, armed forces, help-seeking

## Abstract

**Objectives:**

To explore the accessibility of mental health and gambling related support within the United Kingdom (UK) Armed Forces and investigate potential barriers to engagement.

**Methods:**

We conducted a survey of Armed Forces service personnel (*n* = 438) and specialist healthcare and welfare staff (*n* = 94) regarding gambling harm and related challenges.

**Results:**

Personnel rarely sought on-base support and recognition of existing service provision was low. Among those who did seek help, healthcare and welfare staff were equally effective in engaging with personnel, with greater professional experience playing a key role in fostering meaningful interactions.

**Conclusions:**

Improving access to specialist gambling harm support may enhance help-seeking.

## Introduction

Armed Forces service personnel are at an increased risk of gambling harms relative to the general population.^1,2^ Pritchard and Dymond^3^ found that of 2,119 currently serving members of the Royal Air Force (RAF), 12.5% were at risk of some form of gambling harm. This is significantly greater than the estimated UK general population prevalence of 3.8%.^4^ The high prevalence of gambling harms is not limited to the UK Armed Forces: similar prevalence of gambling harms has been reported in other North Atlantic Treaty Organisation (NATO) structured militaries around the world. Steenbergh et al.^5^ found that of 31,104 US Air Force personnel, 8.1% reported gambling harms, which is almost twice the estimated prevalence in the US general population. Cowlishaw et al.^6^ found high rates of gambling problems (7.7%) among a sample of 1324 Australian personnel post-deployment. Recently, Jones et al.^7^ found that 23% of a sample of *n* = 608 recruited from across the UK Armed Forces reported some level of gambling harm. Risk was particularly elevated among younger, male personnel of lower rank and those experiencing anxiety or post-traumatic stress disorder (PTSD) symptoms. Engagement with strategic gambling activities such as online gambling further predicted harmful gambling.

With accumulating global evidence of elevated gambling risk, structured pathways for treatment and support for UK Armed Forces personnel experiencing harm from gambling remain relatively nascent. In 2023, Defence Primary Healthcare (DPHC)^8^ stated that *“Evidence via disciplinary data, primary care engagement and single Service observation indicates that harm from gambling exists within the military population*” (DPHC, 2023, p.3). The DPHC’s “Delivering an Integrated Gambling Harm Pathway for Primary Care” includes risk-managed links with NHS treatment services for referral to best-practice treatment. Personnel experiencing harm from gambling may receive brief interventions which involve screening, counselling, the provision of feedback, and further signposting to sources of help and support.^9^ Such brief interventions may be delivered by healthcare professionals including nurses, medical officers/doctors, psychologists and psychiatrists who may then make a subsequent referral to the NHS Primary Care Gambling Service (PCGS). However, the uptake and impact of these referral pathways have not been formally evaluated, and routine screening of gambling harm is not yet currently undertaken.

Outside of Defence, the role of third sector support has expanded. For instance, the Armed Forces Gambling Support Network (AFGSN) delivers *Battling the Odds*, a programme raising awareness and building resilience among service personnel and veterans. Other initiatives such as the *Bet You Can Help* programme provides peer-led training and support. These multi-sector developments reflect increasing recognition of military-related gambling harms, acknowledged in recent UK National Institute for Health and Care Excellence (NICE) guidelines for the identification, assessment and management of gambling-related harms.^10^ The guidelines recommend, “*asking people about gambling if they may be at increased risk because of their current or past occupation, for example, armed forces personnel, veterans, …*”. Clearly, considerable work is underway both to establish new gambling services and integrate with existing statutory provision for personnel experiencing harm from gambling.

Research and evaluation of new healthcare service provision often make use of mixed methods designs where both quantitative and qualitative data are gathered. Qualitative research has identified several barriers to accessing the currently available gambling-related support for Armed Forces personnel. Stigma, the cultural normalisation of gambling, and hierarchical attitudes within the military continue to discourage help-seeking, particularly where personnel fear reputational damage or being perceived as weak.^11^ Individuals reported being referred to inappropriate services or facing confusion around the correct point of contact, with access to consistent and relevant care being further compounded by logistical difficulties, such as being based remotely or deployed overseas.^12^ Personnel described a lack of recognition of gambling as a legitimate mental health issue within the Armed Forces and highlighted the need for visible, accessible information about support pathways both on- and off-base.^11,12^

As serving military personnel are considered a group with higher risk of experiencing gambling harm, we conducted a mixed methods investigation employing survey and semi-structured interviews. The present study was part of a wider study concerned with the prevalence and predictors of gambling harm among service personnel.^7^ Our objectives here were to investigate the accessibility and provision of mental health and gambling support within the UK Armed Forces and to identify potential barriers to engagement.

## Materials and methods

### Survey - participants and procedures

Participants were recruited via announcements on the Ministry of Defence DefNet intranet as part of our larger study.^7^ Participation was open to all service personnel, and no attempt was made to ensure representativeness across services, ranks, or gender. The present study therefore included convenience samples of Armed Forces service personnel (AFSP) in a healthcare or welfare support role (H&W; N = 94) and non-H&W role (AFSP; N = 608), respectively. The H&W personnel were described in terms of professional or training background, with respondents reporting a background in healthcare or medicine (e.g., healthcare assistant, nurse, doctor) being grouped as disparate to those reporting a welfare or support work background (e.g., divisional officer, chaplain, case worker). All respondents from the larger study - which included H&W personnel and non-H&W AFSP - answered survey questions regarding sociodemographic characteristics including age, sex, resident country, nationality, ethnicity, educational attainment, relationship status, and current accommodation.

AFSP-only respondents answered questions related to their Armed Forces careers including year of enlistment, rank, salary, specialism, and deployments. AFSP also answered questions regarding past-year gambling, frequency and the types of gambling, and completed mental health measures of mood, anxiety (GAD-2),^13^ post-traumatic stress symptoms (PCL-5),^14^ and loneliness and alcohol questions from the Adult Psychiatric Morbidity Survey.^15^

H&W personnel-only respondents were presented with questions regarding their experience of supporting personnel including how they would describe their role, their typical annual caseload of personnel experiencing gambling harms, and the demographic characteristics of their caseload. They were also asked to list the most common forms of gambling as reported by personnel experiencing gambling harms, as well as the comorbid problems often presented alongside gambling harms. Additionally, H&W personnel were asked whether they had been contacted by concerned others (e.g., friends, colleagues, or family members) by answering along an ordinal scale of 0 (never), 1 (once or twice), 2 (several times) and 3 (many times). They were not asked about their own gambling behaviour.

**Figure 1.**
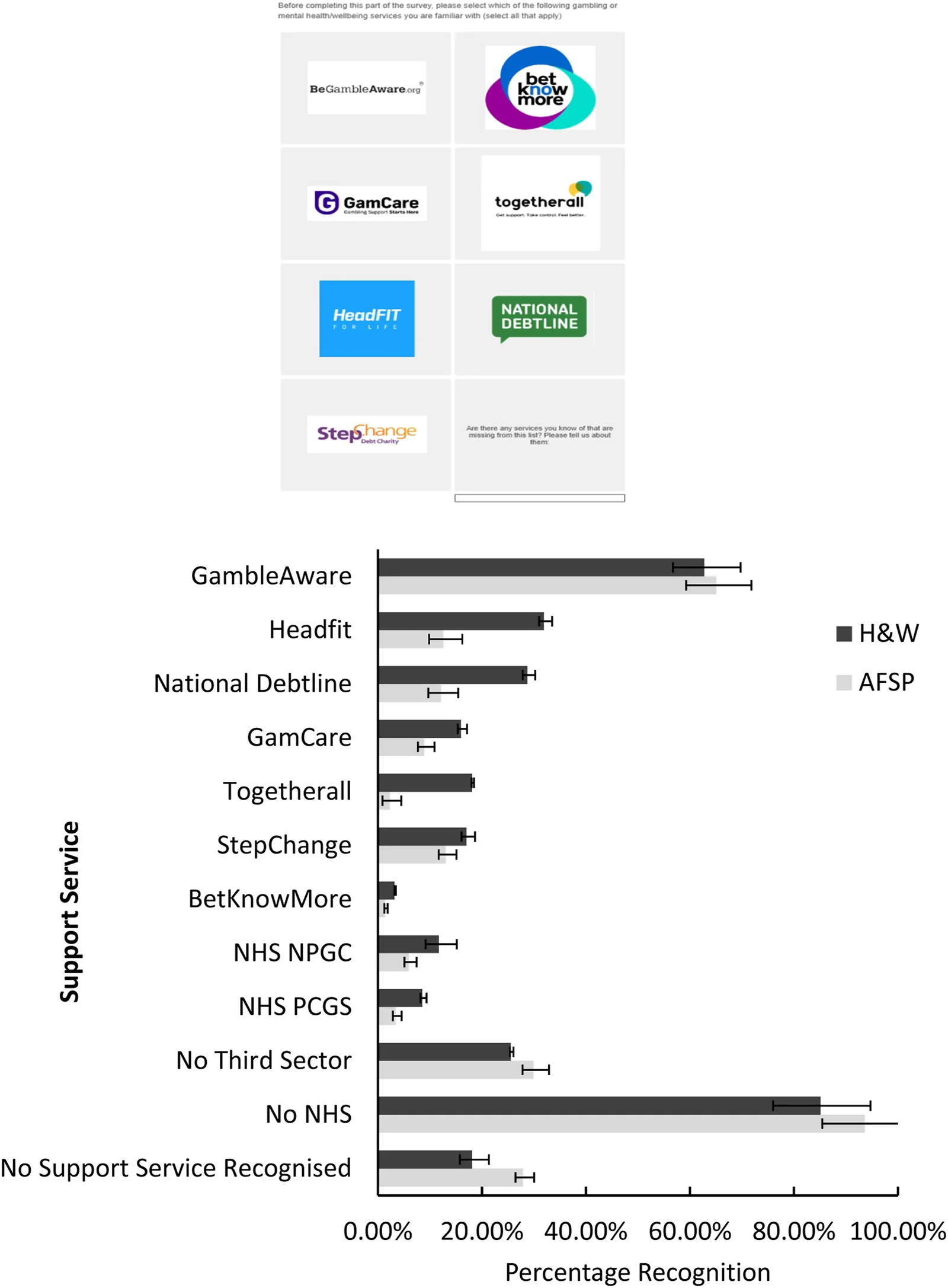
Logo recognition test (upper panel) and performance of those AFSP that had gambled in the past-year and H&W personnel (lower panel). *Note*. “No Third Sector” indicates that none of third sector service providers were recognised, “No NHS” indicates that neither of the two listed NHS treatment service providers (i.e., NHS NPGC and NHS PCGS) were recognised, and “No Support Service Recognised” indicates that none of the support services shown were recognised. Error bars indicate +1/-1 standard deviation.

All respondents (ASP and H&W personnel) then completed a ‘logo recognition quiz’ (Figure 1) to gauge familiarity with the logos of third sector (i.e., GambleAware, Betknowmore, GamCare) and National Health Service (NHS) (i.e., National Problem Gambling Clinic, PCGS) gambling support services, mental health (i.e., Headfit, Togetherall) and financial support services (i.e., National Debtline, StepChange). The logo recognition quiz consisted of the simultaneous presentation of these logos with the instruction to ‘click on all those you recognise or have heard of before’. Participants could also write in “any services you know of that are missing from this list.”

Sociodemographic characteristics included age, sex, educational attainment, service background, living arrangements, and ethnicity are reported for both H&W personnel and AFSP. Non-parametric (Mann and Whitney and Pearson correlation) tests were undertaken, missing data were handled through listwise deletion, and all analysis was carried out using JASP (2025).^16^ Formal adjustments for multiple comparisons were not applied to avoid potentially inflating Type II errors.

### Semi-structured interviews – participants and procedure

Participants who expressed interest in taking part in a follow-up qualitative interview were asked to provide their email address and subsequently sent a participant information sheet and a consent form to read and sign. Participants were offered a choice of conducting their interview over Teams/Zoom, or phone. Eight individuals participated in a follow-up qualitative interview during the autumn and winter of 2023, these each lasted about 60 minutes. The final number of completed interviews was determined by how many individuals expressed interest and could spare the time to take part. The required sample size in qualitative research lacks consensus.^17^ Appropriate sample size for different studies is generally based on researcher judgement. Despite a small sample size, our interviews provided a detailed and rich understanding of an underexplored context for gambling harms. The authors also applied the principle of data saturation, commonly used to determine if a sufficient sample size has been achieved.^18^ In this study, data saturation was reached when no new themes or codes emerged during the final interview.

A semi-structured interview schedule was developed to explore presentations and influencers of gambling and gambling harm in the UK Armed Forces, awareness and education around gambling harm support pathways, and quality of support provision among military health and welfare staff in the context of their roles (see Supplemental Materials).

All participants received a £20 Amazon voucher on completion of the interview. Ethical approval was obtained from the Ministry of Defence Research Ethics Committee (Reference: 2250/MODREC/23).

Interviews were audio recorded and transcribed. For the analysis and reporting, participants were assigned a pseudonym to provide anonymity. Thematic analysis (TA) was utilised to analyse the data.^19,20^ This is a flexible and accessible six-step process which can be used within most theoretical frameworks and to analyse a broad range of qualitative data types. The TA methodology began with the re-reading and note-taking of transcribed data, which allowed HC to start constructing initial thematic concepts. Manual coding and labelling of data segments within each transcript using Excel was then undertaken. These codes were reviewed by HC, MJ, and SD and subsequently merged or collapsed to form developing themes. This was an iterative process which sometimes involved re-visiting transcriptions to ensure that the emerging themes corresponded to the codes and raw data. Once there was agreement with the themes, HC, MJ, and SD named each theme before HC wrote up the results.

## Results

### Survey results

Table 1 shows the survey sample characteristics. A total of 702 responses were received. Of these, 94 were H&W personnel (13.39%), and 438 were non-H&W AFSP (62.39%) reporting past-year gambling. The remaining 170 (24.22%) respondents were AFSP with no gambling in the previous year and were not included in analysis.^7^

**Table 1.** Sample characteristics of the Health & Welfare (H&W) personnel (*n* = 94) and non-H&W Armed Forces Service Personnel (AFSP; *n* = 438).

	Health and welfare (H&W) (*n* = 94)		Armed Forces Service Personnel (AFSP (*n* = 438)	
	*n*	SD/%	*n*	SD/%
Age	40.16	9.26	40.07	9.58
Sex				
Male	49	52.2	374	85.4
Female	45	47.8	50	11.4
Education				
Below A-level	33	35.1	46	10.5
A-level and above	61	64.9	362	82.6
Service branch				
Royal air force	8	8.54	83	18.9
Army	17	18.08	118	26.9
Royal navy	42	44.68	237	54.1
No data	27	28.72	14	3.2

*Note*. Columns may not sum due to incomplete/missing data.

The H&W staff (*n* = 94), who were not asked to report past-year gambling, had a mean age of 40.16 (9.26) years and 14.61% identified as female. The non-H&WAFSP (*n* = 438) who reported past-year gambling, had a mean age of 40 (9.58) years and 85% identified as male.

### Gambling support logo recognition test

Most AFSP with experience of past-year gambling recognised at least one support service (*n* = 307, 70.5%). The most well-known was GambleAware, 65.07% (*n* = 285), and the least known was Betknowmore (*n* = 6, 1.4%). Few personnel recognised either the logos of the NHS National Problem Gambling Clinic (*n* = 26, 5.9%) or the PCGS (*n* = 15, 3.4%), respectively. Notably, 27.9% (*n* = 122) of AFSP did not recognise any gambling support service, whether provided by the third sector, or NHS.

Most H&W staff recognised at least one support service (*n* = 72, 76.6%). Again, the most well-known was GambleAware (*n* = 59, 62.8%), and the least known was Betknowmore (*n* = 3, 3.2%). Compared to AFSP, a minority of H&W personnel recognised either the NHS National Gambling Clinic (*n* = 11, 11.7%) or the PCGS (*n* = 8, 8.5%). Overall, 18.1% (*n* = 17) of H&W personnel did not recognise any gambling support service provider.

Participants could write in “any services you know of that are missing from this list”; however, less than five from each cohort choose to do so.

### Help seeking and engagement

Of the 438 AFSP with experience of past-year gambling, 123 (23%) reported some gambling harm and 6.6% had sought help (*n* = 29). One did so off-base, while the rest did so on-base. There was a small but significant correlation between awareness of support services and help seeking (*r* = 0.16, *p* < .001).

### Educational backgrounds of H&W personnel

Staff with a welfare or support work background self-reported more individuals experiencing gambling harm among their caseloads (*U* = 1219, *p* = .01) with an average of 2 (SD: 1.67), compared with those with backgrounds in healthcare or medicine (1.07; SD: 1.67). They also reported significantly more comorbid difficulties among the people they support experiencing gambling related harm (*U* = 1166, *p* = .04) with a mean of 1.48 (SD: 1.39) compared to 0.94 (SD: 1.38). Welfare and support background personnel also reported significantly more frequent instances of being contacted by concerned others (e.g., friends, family, or colleagues), 0.41 (SD: 0.48) compared to 0.06 (SD: 0.44), (*U* = 1191, *p* = .001).

There were no differences in awareness of gambling support services between those with different backgrounds in terms of the number of qualifications held. However, healthcare and medical workers had a higher average level of qualification of 5.6 (SD 1.87) compared with 4.93 (SD 1.86) for welfare or support (*U* = 679.5, *p* = .027).

### Interview results: UK armed forces H&W support providers’ views of harmful gambling and access to treatment and support

Semi-structured interviews included eight participants from across the Armed Forces, representing a range of welfare, healthcare, and mental health support roles (see Supplemental Materials).

During interviews, four major themes were identified: *(1) Gambling and the role of the military lifestyle, (2) Barriers and delays to prevention, identification and help-seeking, (3) Support pathway obstacles and improvements, and (4) Factors influencing internal support quality*. These themes, accompanying subthemes, and summaries are presented in Table 2.

**Table 2.** Description of themes and sub-themes.

Theme	Summary
1. Gambling and the role of the military lifestyle *Sub-themes* • Culture, conformity, and camaraderie • Unique occupational factors	Military life, and its close-knit group dynamics, deployments, exposure to trauma, boredom, and access to high disposable income, may encourage and normalise gambling
2. Barriers and delays to prevention, identification and help-seeking *Sub-themes* • Minimisation and denial • Stigma, shame, and fear of career repercussions • Improvements to training, education, and overall awareness	Stigma, embarrassment, and fear of being deemed unfit to work discourage and delay personnel from seeking support. Need for investment in safer gambling education, prevention and early intervention
3. Support pathway obstacles and improvements *Sub-themes* • A lack of guidance, clarity, and consistency • The emergence of a standardised pathway	Confusing internal pathways result in inadequate signposting. Yet, it was clear that the advent of the new support pathway does provide a means of assessing, referring, and treating personnel
4. Factors influencing internal support quality *Sub-themes* • A lack of expertise and continuity • The importance of the therapeutic relationship and holistic treatment	Welfare/health staff lack experience of working with personnel with gambling harm leading to reliance on external support services. Deployments, change in posts, and re-locations may hinder support provision. Rapport, trust, and holistic approaches to treatment are key

### Theme 1: Gambling and the role of the military lifestyle

#### Culture, conformity, and camaraderie

Interviewees discussed the impact of Armed Forces culture where “*you’ve got people who associate really closely with each other*” (P7, Psychologist). Gambling traditionally fostered comradeship but also increased the likelihood of personnel gambling if others were doing so - especially when winning:

Close unit cohesion might mean people learn about people's wins and that might not only feed into and perpetuate gambling amongst the unit, but also a gambling addiction with an individual. (P2, Psychologist)

The appropriateness of the Sports Lottery and official raffles were questioned, as they were thought to encourage a culture of gambling within the Armed Forces.

I remember seeing an Aston Martin or one of the high-end sports cars that was on raffle, and it was parked outside Navy Command headquarters. The tickets weren't cheap, in the range of £25 to £100… There was this element of enticing people ‘look what you can win if you've got the money. (P3, Nurse)

This normalisation was regarded as potentially risky and “*unhealthy”* (P4, Doctor).

#### Unique occupational factors

Boredom and loneliness were seen as inevitable in military life, with gambling providing escapism and a way to pass the time, especially for young, single officers:

When they've been deployed overseas, they can gamble online because they are just bored. (P2, Psychologist)

The unique risks and trauma of military life were seen as contributing to complex mental health and addiction issues. Interviewees noted co-occurrences of gambling harm with alcohol addiction, PTSD, and depression, with some also citing links to substance misuse and ADHD.

I do see common other co-morbid problems alongside gambling issues…. Definitely alcohol, and maybe personality traits. And something that’s maybe more of a current thing – substance misuse. (P5, Psychiatrist)

Royal Navy interviewees noted “*a real emphasis on letting loose*” (P4, Doctor) ashore after long periods at sea, leading to extreme gambling and drinking. Such behaviour was rarely seen as problematic (since personnel abstained at sea), but there were concerns it could promote harmful gambling.

I had instances where some of the sailors would spend their entire months wages in one weekend, but then they were going back to sea for another four or six weeks, so it didn't matter too much to them because there wasn't going to be an opportunity to spend their money. (P4, Doctor)

Access to high disposable income at a relatively young age, with little financial education, was seen as increasing the risk of harmful gambling:

One of the things for new individuals joining the military is that they have access to lots of money… And again, because their accommodation and food are provided, they are on a good enough wage that they can just go and spend their money as they wish. (P2, Psychologist)

### Theme 2: Barriers and delays to prevention, identification, and help-seeking

#### Minimisation and denial

Some interviewees had worked with personnel who minimised and denied the extent of their gambling issues. Interviewees thought these barriers to help-seeking were especially common in long-term cases, where individuals had learned to hide their struggles.

I've just had a person recently who I've been seeing for about 2 years for various problems and almost as an aside, when it came to me moving towards discharging them, they mentioned they'd had a very, very significant gambling problem…it shows you that people aren't necessarily keen to come straight out at the get go and say ‘look, by the way also had this gambling issue.’ I think most people minimise. (P5, Psychiatrist)

Harm minimisation was seen as rooted in the normalisation of gambling within the military, where potentially harmful behaviour was dismissed as “*a bit of fun*” (P1, Welfare Officer). As a result, personnel experienced harm while maintaining a ‘*normal*’ life, only seeking support after a pivotal event that “*shocks the individual into recognising what they’re doing*” (P4, Doctor), such as a breakup, work trouble, major financial loss, or arrest for theft.

If other people are around them are doing similar behaviours, they're more likely to just be encouraged along with that sort of group mentality. I don't think they’re educated and informed enough about gambling and the harm that that can bring. (P6, Public Health)

#### Stigma, shame, and fear of career repercussions

Such as being “*medically downgraded*” (P1, Welfare Officer), disciplined, or discharged, were barriers preventing personnel from seeking help. Some line managers also discouraged formal help-seeking, particularly for skilled roles, due to difficulty in finding replacements.

We're an occupational service, so people worry when they come to us for anything that we're going to potentially limit what they do or in some of the more extreme cases recommend that people aren't employed by the forces anymore. (P5, Psychiatrist)

Interviewees agreed that embarrassment and the perceptions of fellow personnel also served as deterrents to help-seeking. Specifically, others knowing that despite working in “*valued and sometimes revered roles*”, they had “*lost money*” and “*procured debt*” (P5, Psychiatrist) would be humiliating. Moreover, disclosing such issues “*would be losing face, admitting weakness*” (P4, Doctor) which is highly stigmatised in military culture.

I do think there still a bit of stigma attached to gambling… people do worry about what people are going to think…the military is still struggling with this mentality of ‘I am strong and therefore any form of admitting any issues, problems or whatever still makes me weak’ and I think that is going to be a very strong stigma to battle down. (P3, Nurse)

Widespread misconceptions among the Armed Forces that gambling harm is not a genuine welfare problem or addiction, and instead something that is a choice that can be controlled was also an issue.

A lot of the military minds about control and I suppose disorders such as this one aren’t necessarily perceived like the other mental health disorders as they’re thought to be a degree of control or greater degree of control and choice over what people are doing. (P5, Psychiatrist)

However, it was widely acknowledged that Defence had begun to focus on improving awareness around gambling harm, which had led to a more open and de-stigmatising culture. Although this shift was “*relatively new*” (P7, Psychologist), interviewees hoped it encourage help-seeking.

I think the awareness of gambling as a potential problem is probably becoming an idea that is just getting to society in general, and I think that it’s also coming into the military more. I think there's something about people being aware that it is potentially a problem and that there are ways of getting help - it's kind of kind of normalisation. (P7, Psychologist)

#### Improvements to training, education, and overall awareness

Gambling-related problems received less attention than other mental health and addiction issues, perpetuating a cycle of inadequate recognition and support. More focus was needed on preventing gambling from escalating and encouraging early help-seeking. It was suggested that the Armed Forces strengthen education and awareness about harmful gambling, incorporating it into mandatory mental health and substance use briefings.

We do have alcohol and drugs awareness and briefings - but I don't think we've captured the gambling in there as well. (P3, Nurse)

Regular, targeted training for line managers and welfare staff was recommended to improve awareness. With suitable training, gambling-related issues could be identified during pre-deployment briefings and screenings if officers knew what to look for. Increased training for the chain of command was hoped to improve buy-in and shift cultural perceptions of gambling as potentially harmful.

I think the support within the day-to-day hierarchy has gaps in terms of awareness and there would benefits from more training or people becoming more aware; it would only help and bring these issues to the forefront. (P1, Welfare Officer)

Several interviewees highlighted that young officers, with large disposable incomes and exposure to gambling, would benefit from financial education during Basic/Phase One Training. One interviewee (P4, Doctor) suggested that the military should provide financial education, covering topics like gambling and financial abuse, to help prevent risky financial behaviours and assist personnel in managing their money effectively.

Several interviewees highlighted the impact of the Addictions and Lifestyles Health Priority Group (HPG), a key part of the Defence People Health and Wellbeing Strategy 2022-2027^21^ as a positive initiative, improving education and understanding of gambling harms.

### Theme 3: Support pathway obstacles and improvements

#### A lack of guidance, clarity, and consistency

Several interviewees noted a lack of clear, efficient support pathways for personnel addressing gambling issues. The consensus was that information on gambling support is poorly publicised and not proactively offered.

It [providing gambling support] is an area that's got a few gaps from my perspective… to help that the best we can we need more of a deepening awareness of what to do if somebody has got a problem. (P5, Psychiatrist)

Some interviewees had encountered difficulties when trying to find relevant internal resources to assist personnel they were supporting and often resorted to web searching external gambling websites. One welfare practitioner said that after spending a considerable amount of time looking for information, they had found a local military support organisation called that they were unfamiliar with:

When I needed resources to help people and I couldn't find much… I used the sort of websites that I could access that provided basic help around gambling care. (P4, Doctor)

A lack of standardised processes detailing what to do if someone discloses a gambling issue was thought to have caused inconsistencies and shortcomings among the response from chain of command. Therefore, line managers’ ability to detect potential issues, advise, and signpost was described as “*quite powerfully individualised, which is a problem*.” (P6, Public Health)

With chain of command, you don't get the same type of response…I don't see any great standardised ‘right, if you come across someone who's showing any signs of gambling harm at all, here's the first three steps to take.’ I think that's probably where the confusion still sits…It needs some attention. (P6, Public Health)

Another challenge was uncertainty over who should be responsible for gambling support, with most interviewees unsure whether it falls under medical or welfare issues. This confusion led to inconsistent approaches and failure to direct personnel to the right support. As a result, Defence medical teams (often GPs) or the Chaplaincy were often the first point of contact, despite not being adequately equipped to handle these issues. Without clear referral pathways, there was pressure on these teams to “*just use whatever knowledge they had personally to manage it*” (P6, Public Health).

I am an armed forces doctor and it’s not inconceivable that someone could ask me that question: ‘Where do I sign post people if they’ve got a gambling problem?’ I have no idea! If they’ve got a problem with alcohol I know the pathway to refer, if they’ve got a clear mental health problem I’ve got a pathway to refer… It feels like [gambling harms] is a real black spot for us - and who's responsibility is this? (P4, Doctor)

#### The emergence of a standardised pathway

Some interviewees noted that gambling harms were becoming part of the UK Armed Forces agenda, citing a new, central multidisciplinary gambling pathway endorsed by the Defence Primary Healthcare Clinical Reference Group.

The main pathway [Gambling Treatment Pathway]…The way that Defence deals with that is to have standard operating procedures that would apply the same guidance across primary care and mental health…There is an effort to get things that are an integrated path that’s running across them so that people are getting the same information, the same guidance about what to do when they come across problems. (P7, Psychologist)

One interviewee was enthusiastic about the pathway’s “*shared care*” component, where PCGS teams keep Defence (particularly the Defence GP) informed of a person’s progress and any ongoing occupational risk, including gambling harm. This was seen as encouraging the military to support personnel in using external gambling services.

This is where it’s (PCGS) different to any other contract we've ever had before and why it's great: they will engage with the GP…. The PCGS retain a relationship with the GP and inform them of any occupational elements that they need to be aware of, and the GP can contact them, so it's a sort of two-way relationship. (P6, Public Health)

### Theme 4: Factors influencing internal support quality

#### A lack of expertise and continuity

The lack of treatment for gambling harms in the military has made it difficult for welfare and health staff to build expertise in this area. Despite strong mental health and addiction teams, the absence of specialists in gambling addiction was concerning.

It's [how to support someone with a gambling issue] a real gap in my knowledge and I often think if that's the case for me that's probably the case for a lot of people. (P4, Doctor)

Crucially, it was believed that this lack of expertise had impacted on the quality of support being offered for those who do decide to seek help for gambling. For example, one interviewee blamed their lack of knowledge in this area on the breakdown of the therapeutic relationship, which ultimately led to a referral to an external gambling support service.

Addiction is not my area of expertise… I did my best with what I had, but eventually I said, ‘look, we need to we need to send you to see an addiction specialist because there's nothing more I can do.’ (P4, Doctor)

A barrier to providing quality internal support was personnel moving posts regularly, which disrupts continuity of care. Being re-posted or deployed often means acquiring a new chain of command, and the new manager may not be aware of the individual’s issues or support. Additionally, the individual may not feel comfortable disclosing their situation to a new manager they have not built a rapport with. There was concern that this disjointedness in communication could “trigger” further gambling harms (P8, Public Health).

People are posted every two years, particularly within the Navy. Say they're being managed within one particular unit for those problems and their chain of command are aware, then they move somewhere else…, is the support actually being continuously provided, and is it being handed over? (P8, Public Health).

Issues with internal support led to an over-reliance on external gambling services. There was concern that external practitioners may not understand the nuances of military life, which can affect treatment. Receiving care from someone who understands both gambling issues and military culture was seen as a crucial aspect of support that is currently lacking.

If we outsource… then we've got a permanent solution, but I've seen it a lot over the last few years where the solutions that are provided to the problems are not armed forces specific and that's a real problem. (P4, Doctor)

#### The importance of the therapeutic relationship and holistic treatment

Despite some shortcomings in internal support, several health and welfare staff interviewees shared examples of providing meaningful support. Rapport and trust were identified as key to successful interventions, particularly for gambling harms. Several practitioners explained that personnel often sought support for other issues and initially hid their gambling problem. It was only after building a therapist-client relationship, sometimes over weeks or months (or even 2 years in one case), that they felt comfortable disclosing it.

For some, they've been referred for a separate problem and after they've built a relationship with me, they then disclose as a follow up a gambling issue. So, they've not come for a gambling issue, they've come for something else - and that might be because gambling isn't the main thing, or it might be because they haven't felt able to share it initially, They've waited until they've been able to share it after they've built a bit of a rapport. (P2, Psychologist)

Providing tailored, holistic support based on individual needs was identified as key to success. Interviewees with experience found a combination of therapeutic and practical interventions, either personally or through external referrals, to be most helpful. Therapeutic support included talking therapy and trauma-based work, while practical support focused on financial aid, safeguarding, and implementing self-exclusion controls to restrict gambling.

It depends on the case, but it can be therapeutic, or more practical stuff…. We look at managing risk and any safeguarding concerns…. For the individual it can be quite regular meetings where they come into the office, or we’ll go out for a walk, and it’s that’s the therapeutic side where we give them the chance to process what’s going on for them. (P1, Welfare Officer)

Finally, interviewees often saw gambling issues co-occur with mental health disorders or addiction. In these cases, they formulated therapeutic support, which reduced the co-occurring gambling harms.

I did get a referral for someone which was around the gambling but actually I said to them that gambling wasn’t the issue, it was trauma and you’re coping with your trauma through gambling. We did an intervention to work on his trauma, which led to a direct reduction in gambling without even specifically looking at the gambling behaviour - it was addressing his guilt and shame about the trauma that led to a massive. (P2, Psychologist)

## Discussion

Our mixed-methods findings capture, for the first time, the lived experience both of service personnel with experience of gambling harm and H&W staff providing treatment and support. They provide insights into help-seeking for gambling treatment among the UK Armed Forces and suggest that there are significant gaps in gambling support awareness, accessibility, and provision.

A major theme identified the perception that military institutional norms encourage behaviours conducive to gambling harm. This aligns with previous research with single-service^11^ and tri-service personnel,^12^ highlighting the normalisation of gambling within military culture. Implications of this include the necessity for military-specific interventions that challenge these norms and integrate discussions about gambling risks and harm-reduction strategies into daily military life.

Various barriers to help-seeking for gambling-related harms were identified. Firstly, issues related to education and awareness were alluded to via both the survey and semi-structured interviews. Most AFSP and H&W personnel recognised the logo of at least one gambling treatment service provider; perhaps unsurprisingly, given its high profile in national advertising and the requirement that it be included in gambling marketing, the logo of GambleAware was the most well recognised. However, both groups demonstrated low recognition of the NHS Problem Gambling Clinic (now the NHS National Gambling Clinic) and the PGCS. Moreover, 28% of AFSP and 18% of H&W personnel, respectively, did not recognise any gambling treatment provider at all. This suggests a broader lack of awareness among staff whose caseloads include service personnel experiencing gambling harm. Increased efforts to promote these services are also necessary to ensure that support networks effectively reach those in need. Currently the evidence regarding gambling harm messaging is limited,^22^ although Gainsbury et al.^23^ argue that education and promotion of such services is required to increase awareness of the resources available, including targeted promotions to increase awareness of relevant services among specific populations.^24^ Secondly, the qualitative findings support prior research indicating that shame and stigma remain a significant barrier to seeking help for gambling harm,^25^ particularly within military settings^12^ and often exacerbated by fears of job-related repercussions.^26,27^ Further, reluctance to seek help was contextualised within the broader hierarchical structures of the Armed Forces, which traditionally emphasise masculine ideas of resilience over emotional vulnerability.^28^ While institutional efforts to reduce stigma were noted, they require systematic implementation to be fully effective. Indeed, H&W interviewees suggested several ways in which internal support mechanisms could be implemented or improved to further help in preventing gambling-related harms addressing deeply embedded, harmful cultural norms around it, and fostering an environment that supports help-seeking behaviours. These included educational campaigns such as financial training during early training and management-level training.^26^ Previous research has also recommended anti-stigma initiatives including “myth-busting”,^26^ and the provision of confidential peer-led support networks^12,24^ to alleviate concerns about help-seeking and its potential career repercussions.

Interestingly, personnel with welfare or support service backgrounds reported encountering more cases of gambling harm than their healthcare and medical counterparts, despite similar levels of awareness regarding available support services. Moreover, interviews indicated issues with H&W personnel lacking expertise and background knowledge in gambling-related problems, thus impeding their ability to sufficiently support service users. Given their exposure to affected individuals, welfare and support service personnel may particularly benefit from targeted educational initiatives.^24,29^ Targeted interventions aimed at improving awareness of support services and training for personnel can enhance help-seeking behaviours.^23,24,26^ Intervention effectiveness may differ across genders, as has been found with mental health help-seeking in the UK Armed Forces,^30^ and lessons learned from comparably low rates of help-seeking among serving and ex-service personnel with self-reported alcohol problems.^31^ Increasing professional exposure, strengthening support networks, developing gender-specific support pathways, and utilising digital interventions^32^ could facilitate greater disclosure of gambling harm among personnel.

Future research should address the following potential limitations. First, recruiting a larger, more representative sample of the AFSP population would facilitate identification of longitudinal trends in gambling harm prevalence, help-seeking, and support. Doing so would also enable a more nuanced understanding of the role of gender in those experiencing gambling harm, particularly as the majority of Armed Forces personnel are male. As such, the present study was limited by the predominantly male convenience sample recruited. In addition, as the present caseload estimates of H&W staff relied on respondents’ memory and may have been unreliable, further studies should employ objective measures wherever possible. Finally, a better understanding of how ethnicity and sexuality shape experiences of gambling harm, as well as comparative international studies, would offer a broader perspective on healthcare service provision for this vulnerable population.

Our findings indicate three primary implications. Firstly, they highlight concrete recommendations for improving education and awareness about gambling harm. Training and support should be enhanced for personnel at all levels, ensuring that service members and management are equipped to provide and access necessary resources. Resources and education should target H&W staff who we have identified as requiring support to be better equipped to work with people harmed by gambling within their caseloads. This includes increasing the visibility of both military and civilian support services. In doing so, there should be further clarity developed for H&W staff to understand where responsibility for support services gambling harm is situated and resourced from in the Armed Forces. At present, there is confusion among the Armed Forces around whether gambling harm is a medical or welfare issue.

Secondly, we recommend implementing educational initiatives across the Armed Forces to enhance visibility and utilisation of both internal and external support services. Such initiatives should emphasise both formal and informal support options, aligning with the broader importance of social support in recovery.^33^ Targeted gambling harm education should be embedded into routine health assessments and screening questions included in the armed forces continuous attitude survey (AFCAS). Doing so would not only contribute evidence to inform future service provision but also foster a culture in which gambling harm is publicly acknowledged and seeking support is normalised.

Thirdly, our findings suggest a broader need for structural and cultural change within the Armed Forces to address gambling harm effectively. Given the normalisation of gambling and the persistent stigma around seeking help, a fundamental shift in military culture and policy is warranted. Similar calls have come from recent work with *n*=28 United States military veterans and service members.^34^ Interviews revealed barriers reflecting military norms which created barriers to help-seeking and inadequate awareness of and access to military-specific support for gambling harm. Such findings mirror those of the present study and provide cross-cultural support for the identification of internal and external barriers to seeking help for gambling related harm in the military. Policy recommendations that arise for this body of work would do well to adopt a public health approach to gambling harm. This approach recognises the broader systemic factors contributing to gambling harm^35^ and may provide a valuable framework for guiding these reforms. To facilitate this, we recommend the adoption of the recently published NICE guidelines,^10^ which include routine screening for gambling harm, targeted assessments for high-risk groups such as service personnel and veterans, the use of standardised screening tools, removing highly addictive slot-machines from military sites, and evaluating strategies to reduce stigma. Furthermore, incorporating destigmatising language throughout military structures^36^ could contribute to a more supportive environment for affected personnel, families and communities.

## Supplemental Material


Supplemental material - Help and support for gambling harm among United Kingdom armed forces personnel: A mixed-methods study
Supplemental material for Help and support for gambling harm among United Kingdom armed forces personnel: A mixed-methods study by Blair Biggar, Hannah Champion, Matthew Jones, Glen Dighton, Justyn Larcombe, Matt Fossey and Simon Dymond in Journal of Health Services Research & Policy

## Data Availability

Data are available from the authors on request.*
