# Supplementary material for: Help and support for gambling harm among United Kingdom Armed Forces personnel: A mixed-methods study
Source: J Health Serv Res Policy. 2025 Dec 20;31(4):255–66. doi: 10.1177/13558196251409041 (PMC13589111; doi:10.1177/13558196251409041)
Supplement: Supplementary file 1 [file sj-pdf-1-hsr-10.1177_13558196251409041.pdf]

Online Supplement

Table S1. H&W staff (*n* = 8) participant information.

Participant	Gender	Service	Role
1	Male	Royal Navy	Welfare worker
2	Female	Tri-service	Psychologist
3	Female	Royal Navy	Nurse
4	Female	Royal Navy	Doctor
5	Male	Royal Air Force	Psychiatrist
6	Female	Tri-service	Public Health
7	Female	Tri-service	Psychologist
8	Female	Tri-service	Public Health

Journal of Health Services Research & Policy
Help and support for gambling harm among United Kingdom Armed Forces personnel: a mixed-methods study
Dymond S, et al

Interview Topic Guide – Healthcare and Welfare Support staff

Interview introduction

Thanks for agreeing to share your experiences of helping armed forces personnel who have experienced harmful gambling. If you would rather not answer a question, then please let me know and we can skip it. You are under no obligation to answer anything you don't want to, or to answer quickly or to give the 'right answer'; you are the expert in your own experiences and that is what we are interested in for this interview.
Also please let me reassure you that your responses remain confidential in taking part in this interview, and though we may include short quotations from interviews in write-ups, no one will be able to identify you from the work we do.

In extreme circumstances where an interviewee states that they or others are at immediate risk of harm due to suicidal or parasuicidal intentions and plans, homicidal intentions and plans, or an inability to carry out high-risk work safely such as operating vehicles or weaponry with the same degree of accuracy or control as would be normally expected of the operator confidentiality will have to be waived, help for the interviewee obtained, and the chain of command notified.

Experience of helping armed forces personnel

- Q1) Could you give me an outline of your role within the armed forces? Prompt: What does your typical working day involve? How often do you see people who are experiencing harmful gambling as part of your usual duties?
- Q2) Do you think the burden of harmful gambling has changed over the time you have worked in your role? Prompt: Do you think the people you support are more or less likely to gamble on base or during leave? If you have been deployed, did you notice that people were more or less likely to reach out for help with gambling when on operations?
- Q3) Do you notice any other problems that commonly occur alongside gambling related problems? Prompt: For example, do people who report issues with their mental or physical health more commonly seek help for gambling too?

Experience of facilitators and barriers to help seeking

- Q4) What do you think helps people reach out when experiencing gambling related harms? Prompt: Do you notice anything that usually prompts people to address their gambling habits, such as pivotal events, encouragement from significant others, friends, or colleagues?
- Q5) Do you think there are any barriers stopping people from seeking help? Prompt: Have you ever noticed barriers such as stigma, fear of harming career, childcare, lack of funds, geographical location, or being unaware of services getting in the way of people seeking help? Do you notice positive or negative attitudes towards help seeking? Do you notice positive or negative assumptions about the help available?

Experience of providing help or signposting

- Q6) In your experience, what kind of exercises or services are helpful for people experiencing gambling related harm? Prompt: Are there any strategies, techniques or exercises you advise for people experiencing harmful gambling? Are there any on or off-base colleagues, services, or websites you often refer people to?

Debrief

Thanks again for helping us better understand the issue of harmful gambling in the armed forces.
